# Large-Area Clay Composite Membranes with Enhanced Permeability for Efficient Dye/Salt Separation

**DOI:** 10.3390/membranes15010025

**Published:** 2025-01-13

**Authors:** Yixuan Fu, Shuai Wang, Huiquan Liu, Ke Zhang, Lunxiang Zhang, Yongchen Song, Zheng Ling

**Affiliations:** 1 Key Laboratory of Ocean Energy Utilization and Energy Conservation of Ministry of Education, School of Energy & Power Engineering, Dalian University of Technology, Dalian 116024, China; fuyixuan7@mail.dlut.edu.cn (Y.F.); wangshuaibbb@126.com (S.W.); lhq971020@mail.dlut.edu.cn (H.L.); zhangke1236@mail.dlut.edu.cn (K.Z.); lunxiangzhang@dlut.edu.cn (L.Z.); songyc@dlut.edu.cn (Y.S.); 2 Ningbo Institute of Dalian University of Technology, Ningbo 315016, China

**Keywords:** two-dimensional nanomaterials, all-clay membranes, assembled membranes from 2D nanomaterials, dye/salt separation, vermiculite

## Abstract

The escalating discharge of textile wastewater with plenty of dye and salt has resulted in serious environmental risks. Membranes assembled from two-dimensional (2D) nanomaterials with many tunable interlayer spacings are promising materials for dye/salt separation. However, the narrow layer spacing and tortuous interlayer transport channels of 2D-material-based membranes limit the processing capacity and the permeability of small salt ions for efficient dye/salt separation. In this work, a novel sepiolite/vermiculite membrane was fabricated using Meyer rod-coating and naturally occurring clay. The intercalation of sepiolite Nanofibers between vermiculite Nanosheets provides additional transport nanochannels and forms looser permeable networks, producing composite membranes with remarkably enhanced flux. As a result, the optimized membranes with 80% sepiolite exhibit remarkable flux as high as 78.12 LMH bar^−1^, outstanding dye rejection (Congo Red~98.26%), and excellent selectivity of dye/salt of 10.41. In addition, this novel all-clay composite membrane demonstrates stable separation performance under acidity, alkalinity and prolonged operation conditions. The large-scale sepiolite/vermiculite membranes made by the simple proposed method using low-cost materials provide new strategies for efficient and environmentally-friendly dye/salt separation.

## 1. Introduction

Global wastewater discharge from the printing and dyeing industry is estimated to exceed 3 billion tons every year [1]. The complex composition and toxicity contained in the highly saline dyeing wastewater is harmful for aquatic ecosystems, human health, and results in the waste of water resources [2]. In addition, salts such as NaCl and Na_2_SO_4_ are added to improve dye uptake in fabric during the dyeing process [3]. The complex wastewater from printing and dyeing industries majorly comprises a high salt content along with the dye, which complicates wastewater treatment strategies and the resourceful recycling and utilization of valuable salts. Various approaches have been developed to treat printing and dyeing wastewater, such as physical adsorption [4,5], electrochemical [6], and biological treatments [7,8]. However, the separation of organic dyes and salts is difficult to achieve by conventional methods [9]. In recent years, nanofiltration (NF) technology has gained popularity among researchers due to its low energy consumption, low operating pressure, and environmental benefits, making it widely used to treat printing and dyeing wastewater [10].

Most of the commercially available NF membranes are made of polymeric materials and exhibit a high rejection of multivalent anions (e.g., sulfate) and organic molecules with molecular weights above 300 g/mol, which reject most of the dye particles and salt ions, hindering the recovery of these salts [11]. For example, a commercial American (Osmonics) NF membrane was used to treat saline dyeing wastewater. However, the membrane exhibited a reactive black 5 rejection of 98.50% and a sodium sulfate rejection of 96.04%, indicating that it rejects both dye molecules and salt ions, which prevents efficient salt recovery and reuse [12]. Thus, it is highly desirable to develop membrane materials that effectively separate salts from dye and facilitate the recycling of resources from printing and dyeing wastewater.

In recent years, NF membranes assembled from two-dimensional (2D) nanomaterials have drawn increased attention owing to tunable interlayer spacing for efficient and selective molecule and ion separation [13]. However, current 2D material membranes (2DMMs) often exhibit low structural stability and limited chemical resistance. Their sensitivity to environmental conditions significantly deteriorates their separation performance due to their low structural stability [14]. Additionally, narrow interlayer spacing and a long, convoluted transport path between Nanosheets limit the flux of 2DMMs. Therefore, it is crucial to find cheap, environmentally friendly, and stabilized 2D membrane material to fabricate NF membranes with a high flux.

Vermiculite is a natural clay mineral with layered structures which can easily be exfoliated into Nanosheets and assembled into membranes for water treatment without the addition of environmentally unfriendly chemicals [15,16]. Vermiculite membranes have shown promising potential in water treatment applications. For example, the nanochannels can selectively transport monovalent ions like Li^+^, Na^+^, and K^+^ while blocking ions such as Cl^−^ and Ca^2+^. The selectivity ratios for Li^+^/Na^+^, Li^+^/K^+^, and Na^+^/K^+^ are 1.26, 1.59, and 1.36, respectively [17]. However, similar to other 2DMMs, the vermiculite membranes also have a limited flux of 26 LMH bar^−1^ [15], limiting their practical utilization. To improve flux in 2DMMs, functionalized nano-intercalators, such as metal–organic frameworks, carbon nanotubes, reduced graphene oxide [18,19,20,21,22,23,24,25], etc., have been used to enlarge the interlayer nanochannels of 2DMMs to promote mass transfer. However, almost all the nano-intercalators are expensive to prepare.

Sepiolite is a natural and low-cost clay mineral with a fibrous structure. When assembled into films and provided additional nanochannels to promote the transportation of water molecules, sepiolite Nanofibers could form loose nanofiber networks that significantly enhance water flux in the as-made composite membranes [26]. Moreover, sepiolite has an abundance of hydrophilic groups on its surface, giving its assembled membranes good hydrophilicity, as well [27]. Thus, sepiolite could be an ideal nano-intercalator to improve desired hydrophilicity and water flux properties.

However, there are several difficulties, particularly scalable fabrication, which must be overcome before nanoclay materials can be practically applied to industrial membrane separation. Existing methods to assemble nanoclay materials such as vacuum-assisted filtration [26,27,28,29] are hard to scale. Therefore, it is important to develop a suitable and large-scale method to assemble large-area clay membranes for dye/salt separation. Several alternative techniques, including spray-coating, drop-casting, and bar-coating, have been shown to effectively fabricate large-area 2DMMs. However, it is hard to fabricate well-ordered lamellar membranes using the spray-coating method [30]. Drop-casting [31] is a discontinuous process that is challenging to scale up for industrial production. The Bar-coating method enables the continuous preparation of large-scale membranes combined with a roll-to-roll deposit process [32]; therefore, it is a potential and promising method in the fabrication of nanoclay membranes. However, there are no reports on the continuous fabrication of all-clay composite membranes using Bar-coating methods.

In this work, we present a new and facile Meyer Bar-coating method using high-concentration clay dispersion with the desired concentration-dependent rheological properties. The composition of the composite membranes can be easily tuned using the clay dispersion. Efficient dye/salt separation was achieved through the simultaneous optimization of abundant permeable nanochannels for molecule separation in vermiculite membranes by the intercalation of sepiolite nanofibers (SNFs). The separation abilities of large-area clay composite membranes for different dyes and salts were investigated. The large-area clay composite membranes exhibited impressive flux, high dye rejection, low salt rejection, and outstanding dye/salt selectivity. The as-made membranes maintained efficient dye and salt separation performance over a wide range of pH, from 2 to 12, and long-term operation conditions. Our eco-friendly films have shown high dye/salt separation ability, high wastewater treatment capacity, and long-term operational stability. This work provides an innovative strategy for the preparation of all-clay composite membranes for highly efficient dye/salt separation.

## 2. Materials and Methods

### 2.1. Materials

Two kinds of natural clay materials, vermiculite particles (with average particle sizes of 1–3 mm) and sepiolite particles, were provided by (Hebei Lingshou Ore Powder Factory Co., Ltd., Shijiazhuang, China). and (Sigma-Aldrich (Shanghai) Trading Co., Ltd., Shanghai, China), respectively. Sodium chloride (NaCl), sodium sulfate (Na_2_SO_4_), hydrochloric acid (HCl), and sodium hydroxide (NaOH) were bought from (Sinopharm Chemical Reagent Co., Ltd., Beijing, China). Methylene Blue (MB) and Congo Red (CR) were obtained from Shanghai Macklin Biochemical Co., Ltd. in Shanghai, China. Polyether- sulfone (PES) (Shanghai Feiling Biotechnology Co., Ltd., Shanghai, China) with an average pore size of 450 nm) was used as the polymeric substrate. Deionized (DI) water, with a resistivity of 18.2 MΩ·cm, was obtained via reverse osmosis in our laboratory.

### 2.2. Preparation of Vermiculite Nanosheet and Sepiolite Nanofiber Dispersions

The procedures used for delaminating vermiculite are reported in our previous work [33]. In a typical run, 3.6 g vermiculite powder (VP) is combined with 100 mL saturated NaCl solution and subjected to hydrothermal treatment at 110 °C for 2 h. Following this treatment, the Na⁺-intercalated VP is separated through vacuum-assisted filtration and thoroughly washed multiple times with deionized (DI) water to remove residual salts. The filter cake is collected and mixed with 100 mL LiCl solution (2 mol L^−1^) and the hydrothermal treatment repeated. The Li⁺-intercalated VP obtained from this process is then dispersed in 240 mL of DI water to facilitate delamination. Liquid-phase delamination is achieved by subjecting the mixture to shear mixing at 20,000 rpm for 20 min. After delamination, the resulting suspension is centrifuged at 300 rpm for 1 h to separate the vermiculite Nanosheet (VNS) dispersion from the sediment, which is discarded. The VNS dispersion is centrifuged at 6000 rpm for 1 h to concentrate it and re-dispersed to achieve a highly concentrated dispersion (20 mg mL⁻^1^) for rod-coating purposes.

A total of 8 g sepiolite powder (SP) was mixed with 400 mL DI water for disaggregation. The liquid-phase disaggregation was conducted by tip sonication for 30 min and then centrifuged at 2000 rpm for 15 min to obtain a SNF dispersion at the desired concentration (20 mg mL^−1^).

### 2.3. Preparation of Sepiolite/Vermiculite Membranes

The as-made VNS and SNF dispersions were mixed at a certain mass ratio using bath sonication. The VNS- and SNF-composite membranes were obtained by Meyer Bar-coating. The Bar-coating technique was conducted at a moving speed of 25 mm s^−1^ using feeding dispersion with a solid concentration of 20 mg mL^−1^. A Meyer Bar (OSP-03) with a groove depth of 3 μm was used to evenly distribute the mixed dispersion onto a PES substrate. Following the coating procedure, the water in the dispersion was promptly evaporated, resulting in the formation of a uniformly coated membrane. All the prepared membranes were air-dried for 30 min to obtain a stable performance. In order to study the composition-dependent separation performance of the Bar-coated membranes, various membranes were fabricated by controlling the mass fractions of SNFs in the VNS/SNF-composite dispersions. The SNF content was controlled at 0 wt%, 20 wt%, 40 wt%, 60 wt%, 80 wt%, and 100 wt% in the VNS/SNF-composite membranes. The membranes were denoted as SVM-*x*, where *x* represents the mass fractions of SNFs in the VNS/SNF-composite membranes. The mass loading of the produced membranes was kept constant at 0.15 mg cm^−2^.

### 2.4. Characterization

The viscosity measurement of different dispersions was carried out using an advanced rheometer (TA-AR2000ex, TA instruments, New Castle, DE, USA) at 25 °C. The surface and cross-sectional morphologies of the as-made membranes were analyzed by scanning electron microscopy (SEM, SU8220, Hitachi High-Tech Corporation, Beijing, China, operated at 5.0 kV and 10 μA). The crystal structures and compositions of the membranes were analyzed using X-ray diffraction (operated at 40 kV and 40 mA; CuKα radiation, λ = 0.15418 nm; 2θ ranging from 3° to 80° with a step 1.5° min^−1^; Bruker D8 Advanced powder XRD, Bruker Beijing Scientific Technology Co., Ltd., Beijing, China) and Fourier transform infrared spectra (FT-IR, Nicolet 6700 instrument with a wavenumber range of 4000–400 cm^−1^ and a resolution of 4 cm^−1^). The small-angle X-ray scattering (SAXS) measurements were carried out at ambient temperatures using Nanopix-3.5 (operated at 40 kV and 30 mA; CuKα radiation, λ = 0.15418 nm; 2θranging from 0.031° to 4.1°, Rigaky Corporation, Tokyo, Japan). Contact angles were measured using an industrial camera (UI-1220LE-M-GL, IDS-Germany, Ningbo, China) and SurfaceMeterTM software 3.15 (Ningbo NB Scientific Instruments Co., Ltd., Ningbo, China), with a 12 μL water drop volume for each test. The zeta potential of membranes in mediums of different pH was tested using the solid-surface zeta potential analyzer (Anton Paar (Shanghai) Trading Co., Ltd., Shanghai, China) by streaming potential measurement.

### 2.5. The Evaluation of Membrane-Separation Performance

The separation performance of the membranes was characterized using a vacuum-filtration unit with an effective area (*A*) of 9.752 cm^2^. The flux *M* (L m^−2^ h^−2^ bar^−1^, also labeled as LMH bar^−1^) of the as-made membranes was evaluated by measuring the permeated water (*V*) during a certain time (*t*) under a specific pumping pressure (*P*), and was calculated using Equation (1), as follows [22]:

(1) $$M=\frac{V}{P\times t\times A}.$$

Different types of mimical wastewater containing dye/salt mixtures were used to describe the separation performances of the membranes. The dye concentration of feed and permeate solutions was measured by a UV–vis spectrophotometer (Lambda 750) and the maximum absorption wavelengths were 490 nm and 665 nm to identify CR and MB, respectively. In addition, the concentration of the salt solutions was determined by measuring their conductivity using an ion conductivity meter (ET915, eDAQ TECH, Sydney, Australia). For solutions containing both salts and dyes, an Inductive Coupled Plasma Emission Spectrometer (PerkinElmer Avio 500 ICP-OES, Shanghai, China) was used to test the cation content. The filtered dye/salt solution was taken and placed into a crucible and heated with an alcohol lamp until the water completely evaporated and the salt crystalized during evaporation. All the salt crystals were collected for characterization. The rejection ratio (*R*) was determined based on the concentration of the permeate (*C_p_*) and feed solution (*C_f_*), as described in Equation (2), as follows [34]:

(2) $$R(\%)=(1-\frac{C_{p}}{C_{f}})\times 100.$$

The separation factor (*S*) was calculated based on the rejection of salt (*R_salt_*) and dye (*R_dye_*), using Equation (3), as follows [35]:

(3) $$S=\frac{R_{salt}}{R_{dye}}$$

### 2.6. Membrane Stability Evaluation

To study the stability of the as-made membranes in a solution, several pieces of membrane (1 cm × 3 cm) were submerged in various aqueous solutions, including DI water, acidic solution (0.1 M HCI, pH = 1), and alkaline solution (0.1 M NaOH, pH = 13) for 48 h. Moreover, the long-term operation stability of the SVM-*x*s was evaluated through 24 h of continuous filtration with a positive-pressure filter device (SUS304, Haining Kezhun Filtration Equipment Co., Ltd., Haining, China). The flux and rejection of the dye/salt mixture (1 g L^−1^ NaCl and 10 mg L^−1^ MB) were tested to study their time-dependent separation performance.

## 3. Results and Discussion

### 3.1. Fabrication and Characterization of the SVM-xs

**Figure 1:**
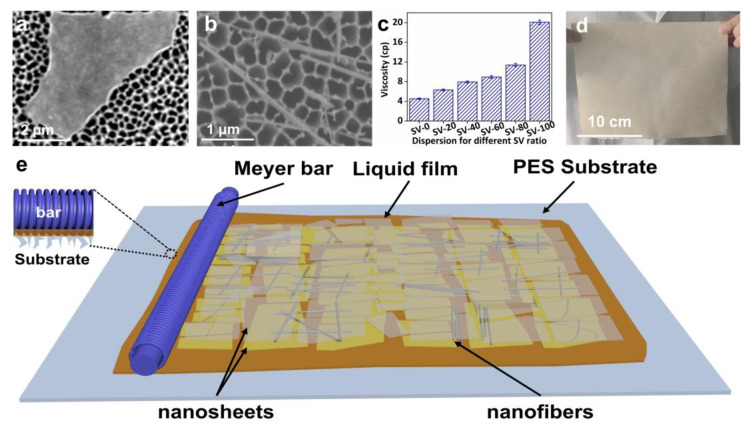
SEM images of (**a**) VNSs and (**b**) SNFs. (**c**) Viscosity of dispersion of different mass ratios of SNFs. (**d**) Digital image of large-area SVM-80 (size: 20 cm × 30 cm; load: 0.15 mg cm^−2^). (**e**) A diagram illustrating the fabrication processes of the SVM−*x*s.

We obtained VNSs and SNFs as building blocks using liquid-phase exfoliation and dispersion. The as-synthesized VNSs exhibited a large lateral size, reaching several micrometers, as shown in Figure 1a. SEM analysis of over 300 Nanosheets revealed the lateral size distribution of the VNSs was centered around 3.77 μm (Figure S1). The as-made SNFs exhibited a typical one-dimensional structure, as shown in Figure 1b, which may circumvent problems such as severe stacking in VNSs. The length and diameter of more than 300 dispersed SNFs were recorded. The average length and diameter of the SNFs were 2.47 μm and 18.27 nm, respectively (Figure S2). As shown in Figure 1c, a piece of large-area (20 cm × 30 cm) SVM-80 was fabricated using Meyer Bar-coating to demonstrate the potential of this proposed method for large-scale practical application. The composition of the all-clay composite membranes can be easily tuned by controlling the mass ratio of the used SNF and VNS dispersions for Meyer Bar-coating. Figure 1d shows the effect of the mass fraction of SNFs on the viscosity of the SNF/VNS-composite dispersions. The viscosity increases with increases in the mass fraction of SNFs in the composite dispersions, producing the desired rheological behaviors for Bar-coating. However, the pure SNF dispersion has a viscosity of 20.08 cp, which is too viscous to form intact membranes, as demonstrated in Figure S3. As schematically shown in Figure 1e, the SNF/VNS dispersions were coated onto a porous PES substrate and the 2D VNSs were assembled into films via interlocking under shearing action due to their large lateral size and ultrathin thickness [36].

**Figure 2:**
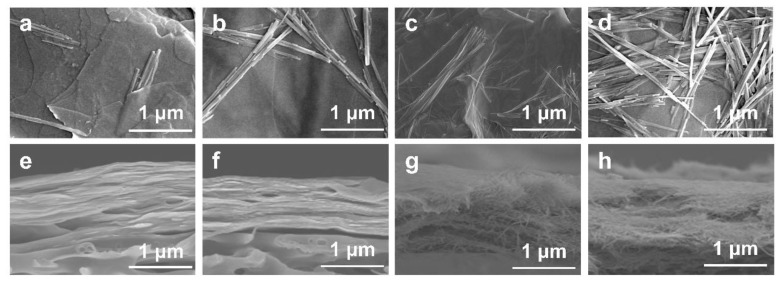
(**a**–**d**) Top-view SEM images of SVM−20, SVM−40, SVM−60, and SVM−80. (**e**–**h**) Cross-section SEM images of SVM−20, SVM−40, SVM−60, and SVM−80.

Top-view and cross-section SEM images of the SVM-*x*s are presented in Figure 2a–h. The number of SNFs on the surface and cross-section increases with an increase in SNFs’ mass ratio. It is worth noting that the VNSs still maintained a stacked structure; even the SNF content in SVM-80 was 80 wt%. The loose structure of the SVM-*x*s could increase flux in the as-made membranes, while the stacked structure helps to reject large molecules such as dyes. These designed composite membranes are suitable for efficient dye/salt separation.

**Figure 3:**
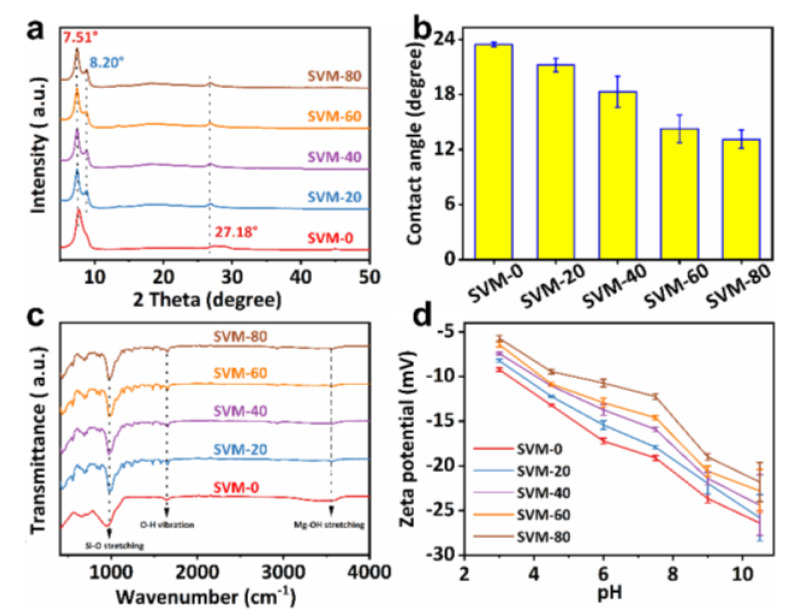
(**a**) The XRD patterns, (**b**) water contact angles, (**c**) FT-IR spectra, and (**d**) pH and composition-dependent zeta potential of the SVM−*x*s.

XRD was used to analyze the structure of the as-made membranes. As shown in Figure 3a, the characteristic peaks at 7.50° and 27.18° can be indexed to (002) and (008) planes of vermiculite, respectively [37]. The increased value of d_002_ from 1.01 up to 1.19 nm, compared with the original vermiculite powders, is due to the intercalation of water during assembly of the delaminated VNSs [33]. Moreover, the characteristic peak at 8.20° is indexed to the (110) plane of the crystalline sepiolite, confirming the successful introduction of SNFs [38]. In addition, the introduction of SNFs did not change the characteristic peaks and half-peak widths corresponding to the vermiculite lattice planes of (001). These results imply that the introduction of sepiolite did not change the interlayer distance and stacking regularity of the VNSs, which was also confirmed by the SAXS (Figure S4). FTIR spectroscopy was also conducted on the SVM-*x*s to analyze the chemical composition of the as-made membranes. The water contact angle (WCA) was used to evaluate the composition-dependent surface hydrophilicity of the as-made composite membranes. As shown in Figure 3b, it can be seen that the WCAs of the composite membranes decrease considerably with the content of the added SNF in the membranes. Especially, the equilibrium value of the SVM-80 membrane decreased to as low as 13.1°, indicating its improved hydrophilicity. SVM-80 exhibits the best hydrophilicity due to it having the highest SNF mass ratio. It can be explained by the ability of the SNFs to adsorb water via the functional groups of Si-OH and Mg-OH [26].

Figure 3c reveals that the two bonds at 1631 cm^−1^ and 980 cm^−1^, which appear in all samples, correspond to the O-H bending vibration and Si-O stretching vibration [39]. Compared to SVM-0, the addition of SNFs introduced a bond at 3666 cm^−1^ which is attributed to Mg-OH stretching in the octahedral layer [40]. The introduction of Mg-OH can enhance the wettability of the SVM-*x*s, which may affect their water treatment capability.

The Donnan effect indicates that the surface zeta potential of a membrane plays a crucial role in determining its separation and rejection properties, particularly for ion sieving [41]. Figure 3d displays the negative zeta potential of all the as-prepared SVM-*x*s, varied by their different pH values, which are attributed to the negative charge of VNSs and SNFs (as shown in Figure S5). The zeta potential of the as-made membranes decreases in absolute value with an increase in SNFs’ mass fraction, which would be helpful for ion/dye separation.

### 3.2. Separation Performance of the SVM-xs in Different Dyes and Salts

**Figure 4:**
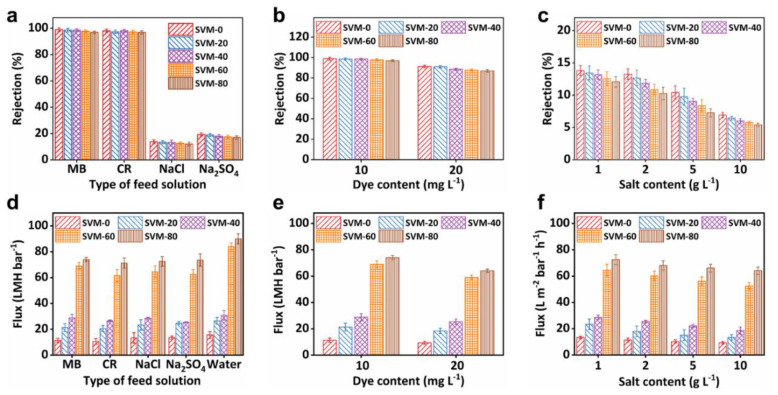
Rejection and flux of the SVM−*x*s: Rejection varied with the (**a**) type of feed solution, (**b**) different dye (MB) content, and (**c**) different salt (NaCl) content. Flux varied with the (**d**) type of feed solution, (**e**) different dye (MB) content, and (**f**) different salt (NaCl) content.

Two dyes (CR and MB) and two salts (NaCl and Na_2_SO_4_) were used to investigate the separation performance of the SVM-*x*s. As revealed by Figure 4a, the dye rejection of the SVM-*x*s was above 96.50% while the salt rejection was below 19.25%, regardless of the composition of the as-made composite membranes, making the SVM-*x*s suitable for dye/salt separation. The rejection of the SVM-*x*s in each pair of dye and salt decreased slightly with an increase in the SNF content. For example, as the SNF concentration increased from 0% to 80%, the MB rejection of the SVM-*x*s decreased by 1.99% (from 98.87% to 96.90%). The NaCl rejection of the SVM-*x*s decreased by 6.87% (from 13.82% to 12.87%). The addition of SNFs to the network caused the clay composite membranes to become looser, thereby facilitating the passage of dye molecules and ions through the membranes. Figure 4b,c shows the dye and salt concentration-dependent rejection of the SVM-*x*s. As the dye and salt concentration increased, the dye rejection of SVM-80 decreased slightly (by 7.79% in MB) while salt rejection decreased rapidly (by 50.12% in NaCl). The ratio of dye rejection to salt rejection, corresponding to the separation factor, increased from 8.02 to 16.06 (an increase of 100.24%). This demonstrates that the membranes enhanced the differentiation between dye and salt rejection, favoring the separation of dye and salt by tuning the membranes’ composition.

The flux variation of the SVM-*x*s was also investigated. It is known that the flux of general nanofiltration membranes is less than 60 LMH bar^−1^ because of their tight structure [9]. As shown in Figure 4d, the flux of the SVM-*x*s increased remarkably with an increase in the SNF mass ratio due to extra nanochannels from the intercalated SNFs. As the SNF concentration increased from 0% to 80%, the flux of the SVM-*x*s increased by 278.24%, from 22.15 LMH bar^−1^ to 83.78 LMH bar^−1^, which improved the efficiency of water treatment significantly, while the high rejection of SVM-80 was maintained above 96.57%. The increased flux of SVM-80 can be attributed to two factors. On the one hand, the network formed by the SNFs makes the clay composite membranes looser, facilitating water molecules and ions to pass through the SVM-*x*s. On the other hand, SNFs replace some of the VNSs to form looser cross-networks to provide additional nanochannels. These additional nanochannels provide more pathways for the transport of salt ions and water molecules which increase flux. In addition, the rejection of CR is nearly identical to the rejection of MB (96.90% vs. 96.83% in SVM-80) while the rejection of Na_2_SO_4_ is far higher than that of NaCl (16.93% vs. 12.07% in SVM-80). This can be explained in terms of sieving and dielectric [42] exclusion effects. The hydrated ionic radius of salts were in the following order: SO_4_^2−^ (0.38 nm) > Na^+^ (0.36 nm) > Cl^−^ (0.33 nm) [43]. Moreover, the negatively charged SVM-*x*s likely generated stronger electrostatic repulsion in bivalent ions (SO_4_^2−^) than in monovalent ions (Cl^−^) [44]. Consequently, the combined effects of sieving and dielectric exclusion led to a higher rejection of Na_2_SO_4_ than NaCl.

As shown in Figure 4e, the flux of dye (MB) reduced with an increase in dye concentration. As the MB concentration increased from 10 to 20 mg L^−1^, the flux of SVM-80 decreased by 13.38%, from 74.03 LMH bar^−1^ to 64.12 LMH bar^−1^. Flux decline is known to be caused by the deposition of dye within the membrane’s pores or onto its external surface [44]. As shown in Figure 4f, the flux of NaCl solutions slightly reduced with an increase in the salt concentration. As the salt concentration increased from 1 to 10 g L^−1^, the flux of SVM-80 demonstrated a negligible decrease of 4.81%, from 72.05 LMH bar^−1^ to 68.89 LMH bar^−1^, due to salt concentration polarization hindering water permeation. Overall, SVM-80 still maintained a high flux (>64.10 LMH bar^−1^) when used to treat higher concentrations of salt and dye.

In brief, the SVM-*x*s had weak salt rejections below 19.50% but high dye rejections above 96.50%. In addition, salt rejection decreased much more rapidly than dye retention with an increase in feed concentration. The significant difference between the dye and salt rejections of the SVM-*x*s indicates their immense potential for the effective separation of dyes and salts. Additionally, the composite membranes also show an impressively high water flux of 84.03 LMH bar^−1^, suggesting their practical application in the treatment of polluted water.

### 3.3. The Separation Performance of SVM-xs in Dye/Salt Solutions

**Figure 5:**
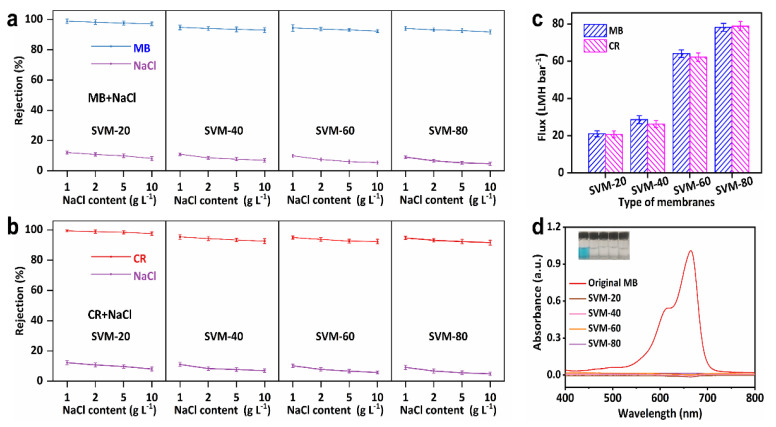
The filtration performance of dye/salt mixtures using the SVM−*x*s (all dye contents are 10 mg L^−1^, all NaCl contents are 1 g L^−1^). (**a**,**b**) NaCl concentration-dependent dye and salt rejections. (**c**) Flux of dye/salt mixtures of the SVM−*x*s. (**d**) The UV-vis spectra of the MB/NaCl solutions before and after treatment using the SVM−*x*s.

Dyeing wastewater usually contains considerable amounts of salt; therefore, it is necessary to investigate separation performance when treating dye/salt mixtures. As shown in Figure 5a,b, all the as-made SVM-*x*s had very high dye rejection (>96.50%) when used to treat dye/salt mixtures, and demonstrates a negligible decrease, from 98.71% to 97.12%, with an increase in the NaCl concentration, from 1 g L^−1^ to 10 g L^−1^. It is known that salts can interact with charged dye molecules, promoting a more uniform dispersion [44]. Therefore, dye particles in dye/salt solutions are likely to pass through the membranes more easily than those in pure dye solutions, leading to lower dye rejection. Furthermore, the salt rejection of SVM-80 was low (<15%) and demonstrated a more significant decrease with an increase in salt concentration due to a larger concentration gradient across the membrane and a reduced Debye length, which weakened electrostatic interactions between the charged ions and the negatively charged membrane surface [45]. As shown in Figure 5c, although the SVM-*x*s have a similar rejection for dyes and salts, SVM-80 has a 274.60% higher flux (78.78 LMH bar^−1^ in the MB/NaCl mixture and 78.12 LMH bar^−1^ in the CR/NaCl mixture) than SVM-20 when used to treat dye/salt mixtures (21.03 LMH bar^−1^ in MB/NaCl and 19.89 LMH bar^−1^ in CR/NaCl). Thus, SVM-80 is the better choice for dye/salt separation due to its outstanding flux and high rejection performance. Figure 5d and Figure S6 confirm the high dye rejection of the SVM-*x*s by UV-vis spectra and optical photos of dye solutions before and after filtrating treatment.

A comparison of the dye/salt separation performances of the membranes assembled from other 2D materials with nano-intercalators are summarized in Table 1 and Table S1 [20,21,22,25,46,47,48,49,50,51,52]. Clearly, the permeation flux (78.12 LMH bar^−1^) of SVM-80 was significantly higher than in most of the reported membranes, confirming SVM-80 can rapidly treat a large amount of salt-containing dye wastewater. Furthermore, SVM-80 also has a superior dye/salt selectivity of 10.41. It is worth noting that both sepiolite and vermiculite are natural minerals and are inexpensive, easy to prepare, and suitable for large-scale assembly. The above-mentioned merits make the practical application of these all-clay composite membranes promising.

### 3.4. SVM-x Membrane Stability

**Figure 6:**
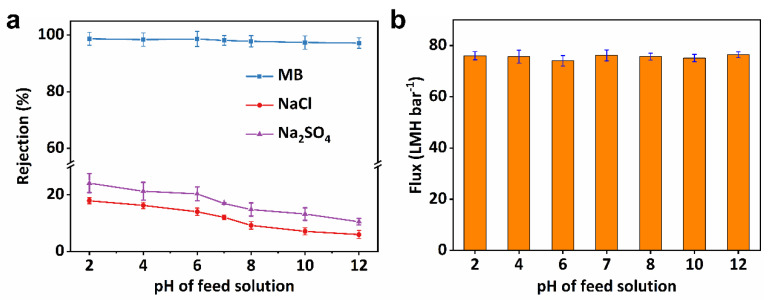
The influence of pH on dye/salt separation. The pH-dependent (**a**) rejection and (**b**) flux data of SVM−80, used for the separation.

Due to its having the best flux and outstanding separation performance, SVM-80 was chosen to study the operation of its condition-dependent separation performance to further confirm its practicality. As textile wastewater is mostly characterized by its high pH (i.e., 6–10) [10,54], especially in industrial dyeing effluent, which contains NaCl or Na_2_SO_4_, the effect of pH on separation performance was investigated. Charge effects have a significant impact on salt ion exclusion, as salt rejection changes significantly at different pHs [52]. Figure 6a shows the influence of solution pH on the rejection of NaCl and Na_2_SO_4_. Na_2_SO_4_ rejection decreased from 24.12% to 10.52% with an increase in pH from 2 to 12. The same trend was observed in the removal of NaCl. However, the NaCl rejection was obviously lower than that of Na_2_SO_4_ due to the low charge density of Cl^−^ compared to SO_4_^2−^. Due to the selective removal of dye rather than salt, SVM-80 showed an impressive selectivity of 7.03~13.68 in pH ranges between 6 and 10, confirming the practicality of this all-clay composite membrane in treating dyeing effluent. As shown in Figure 6b, flux remained almost intact with an increase in the pH of the feed solutions. Although the zeta potential of both MB and the membrane surface was affected by a solution’s pH [55], SVM-80 still exhibited an excellent ability to retaining MB. Dye rejection remained stable over a wide range of pH, from 2 to 12, suggesting the predominance of size-sieving effects in dye removal [2].

**Figure 7:**
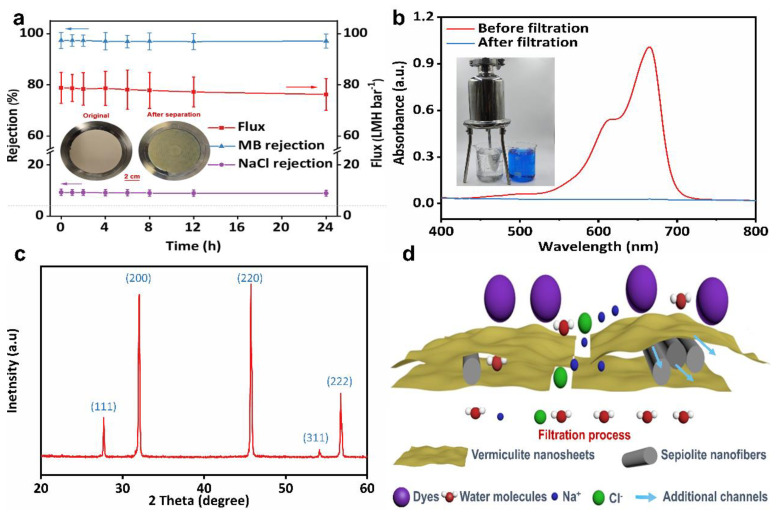
(**a**) The long-term stability and dye/salt separation of SVM−80. The simulated wastewater used in testing contained 1 g L^−1^ NaCl and 10 mg L^−1^ MB. The insets show the outstanding stability of SVM-80 after long-term separation. (**b**) The UV−vis spectra of MB/NaCl solutions before and after long−term filtration using SVM-80. The inset shows a digital image of the scalable MB removal using SVM-80 and a positive−pressure filter. (**c**) The XRD patterns of the recovered salts after filtration and evaporative crystallization. (**d**) A diagram illustrating the dye/salt separation processes of the SVM−*x*s.

Figure 7a shows the performance data of SVM-80 for the large scale and continuous treatment of a MB/NaCl mixture over 24 h. Due to the stable nanochannels and excellent hydrophilicity of the all-clay composite membrane, the flux remained at a high level of 75–80 LMH bar^−1^ with almost intact dye/salt selectivity (9.76–10.41) over 24 h of operation. All the sepiolite/vermiculite membranes showed good stability after immersion in solutions of a different pH for 7 days, as indicated in Figures S7 and S8. As shown in Figure 7b, the MB/NaCl mixture was filtered through a scalable positive-pressure filter using SVM-80 as the filter membrane. The filtered water shows no residual dye, confirming the practical application of the as-made clay composite membrane. To recover salt from the dyeing wastewater, we evaporated and crystallized the filtered solution. Figure 7c shows the XRD pattern of the recovered salts. The five characteristic peaks at 27.45°, 31.79°, 45.53°, 53.94°, and 56.57° correspond to the (111), (200), (220), (311), and (222) planes of NaCl, respectively [56]. The sharp and well-defined peaks confirm a high degree of crystallization and purity of the NaCl crystals obtained from the product. Figure 7d schematically shows the dye/salt separation processes of the SVM-*x*s. SNFs were sandwiched between assembled VNSs, preventing the severe stacking of VNSs and formation of loose lamellar structures. The SNF-intercalated lamellar structures have additional nanochannels compared to the membranes with only VNSs (as shown in Figures S9 and S10). The loose lamellar structures and additional nanochannels contribute to the excellent dye/salt separation performance of the SVM-*x*s applied to large-scale and continuously treated dyeing wastewater.

## 4. Conclusions

Large-area all-clay composite membranes were designed and fabricated through Meyer Bar-coating using natural clay Nanofibers and Nanosheets as building blocks. The intercalation of sepiolite Nanofibers provided additional transport nanochannels and constructed a looser permeable network for the composite all-clay membranes, which showed remarkably enhanced flux due to the formed transport nanochannels. The optimal membrane, with 80 wt% sepiolite (SVM-80), exhibited impressive flux (78.12 LMH bar^−1^), high dye rejection (CR~98.26%), low salt rejection (NaCl~9.44%), and outstanding dye/salt selectivity (10.41). Even in wide ranges of pH, from 2 to 12, SVM-80 maintained a high dye rejection and low salt rejection. Additionally, large flux, outstanding dye rejection, and excellent dye/salt selectivity were maintained during the 24 h continuous filtration test, confirming its excellent separation performance in long-term operation conditions. The all-clay composite membranes were made of green and low-cost materials, based on a simple, yet scalable, preparation method. These low-cost, easily prepared, and scalable membranes with high flux and dye/salt selectivity can serve as potential candidates for the efficient treatment of dyeing wastewater in an industrialized and environmentally friendly way.

## Figures and Tables

**Table 1:** Comparison of separation performance of 2DMMs with nano-intercalators.

Materials	Dye Rejection (%)	NaCl Rejection (%)	Flux (LMH bar^−1^)	Separation Factor	Ref.
MXene/PRGO	100 (CR)	5.3	48.6	18.87	[25]
MXene/MoS_2_	95 (CR)	4.2	154	22.62	[50]
MXene/ZIF-8	99.7 (CR)	7.8	40.8	12.78	[21]
MXene/CNT	99 (CR)	23.5	10.8	4.21	[51]
GO/MoS_2_	99.6 (CR)	43.2	10.2	2.31	[48]
GO/CNTs	98.7 (CR)	3.1	26.3	31.84	[22]
GO/HNTs	97.9 (RB5)	14.3	11.3	6.85	[47]
GO/NH_2_-Fe_3_O_4_	94 (CR)	15	78	6.27	[20]
GO/SiO_2_	99.99 (CR)	4.92	70.7	20.32	[53]
Vermiculite/ Sepiolite	98.26 (CR)	9.44	78.12	10.41	This work

## Data Availability

The raw data supporting the conclusions of this article will be made available by the authors on request.

## Supplementary Materials

The following supporting information can be downloaded at: https://www.mdpi.com/article/10.3390/membranes15010025/s1, Figure S1: Lateral size distribution (300 sheets analyzed) of VNSs obtained from SEM; Figure S2. Length and diameter distribution (300 fibers analyzed) of SNFs obtained from SEM; Figure S3. Digital images of blank PES membrane the membranes fabricated by SVD-100 (pure sepiolite dispersion) using Meyer rod coating; Figure S4. SAXS patterns of SVM-x; Figure S5. Concentration-dependent Zeta potentials of VNSs and SNFs dispersion; Figure S6. The UV-vis spectra of CR/NaCl solutions before and after treatment using SVM-x; Figure S7. The digital photos of SVM-X before and after soaked in solutions of different pH; Figure S8. 84 h stability and dye/salt separation tests of SVM-80; Figure S9. SEM images of SVM-0; Figure S10. Pore size of SVM-0 and SVM-80 measured by the mercuric depressor. Table S1: Comparison of salt retention capacity of other nanofiltration membranes. All the salts are 1 g/L NaCl [20,21,25,46,46,47,48,49,51].
