# Supplementary material for: Large-Area Clay Composite Membranes with Enhanced Permeability for Efficient Dye/Salt Separation
Source: Membranes (Basel). 2025 Jan 13;15(1):25. doi: 10.3390/membranes15010025 (PMC11767988; doi:10.3390/membranes15010025)
Supplement: Supplementary file 1 [file membranes-15-00025-s001.zip › membranes-3367260-supplementary.pdf]

Supporting information

Large-area clay composite membranes with enhanced permeability for efficient dye/salt separation

Yixuan Fu[1], Shuai Wang[1], Huiquan Liu[1], Ke Zhang[1], Lunxiang Zhang[1,2], Yongchen Song[1,2], Zheng Ling[1,2*]

[1]Key Laboratory of Ocean Energy Utilization and Energy Conservation of Ministry of Education, School of Energy & Power Engineering, Dalian University of Technology, Dalian 116024, China

[2] Ningbo Institute of Dalian University of Technology, Ningbo, 315016, China

*Corresponding authors. Tel.: +86 0411 84708015

E-mail address: zling@dlut.edu.cn (Z Ling)

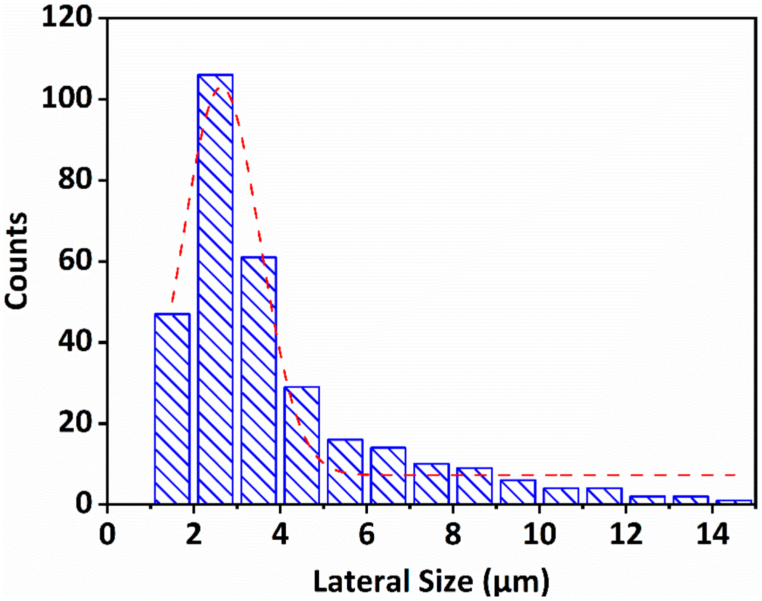

Figure S1. Lateral size distribution (300 sheets analyzed) of VNSs obtained from SEM.

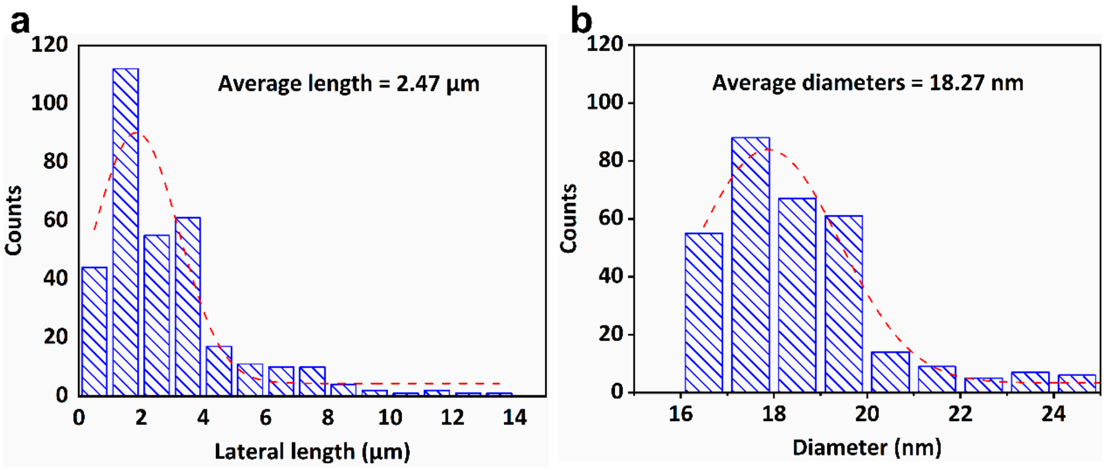

Figure S2. (a) Length and (b) diameter distribution (300 fibers analyzed) of SNFs obtained from SEM.

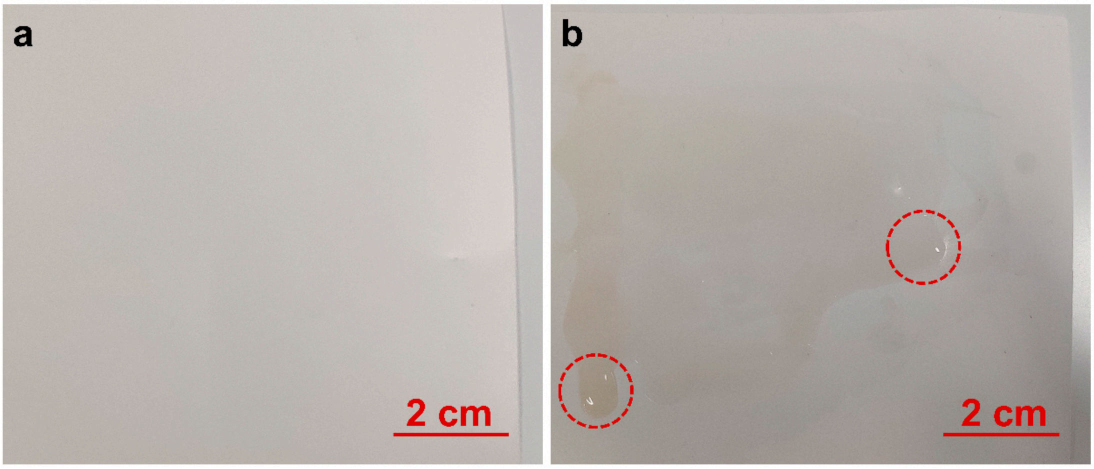

Figure S3. Digital images of (a) blank PES membrane (b) the membranes fabricated by SVD-100 (pure sepiolite dispersion) using Meyer rod coating.

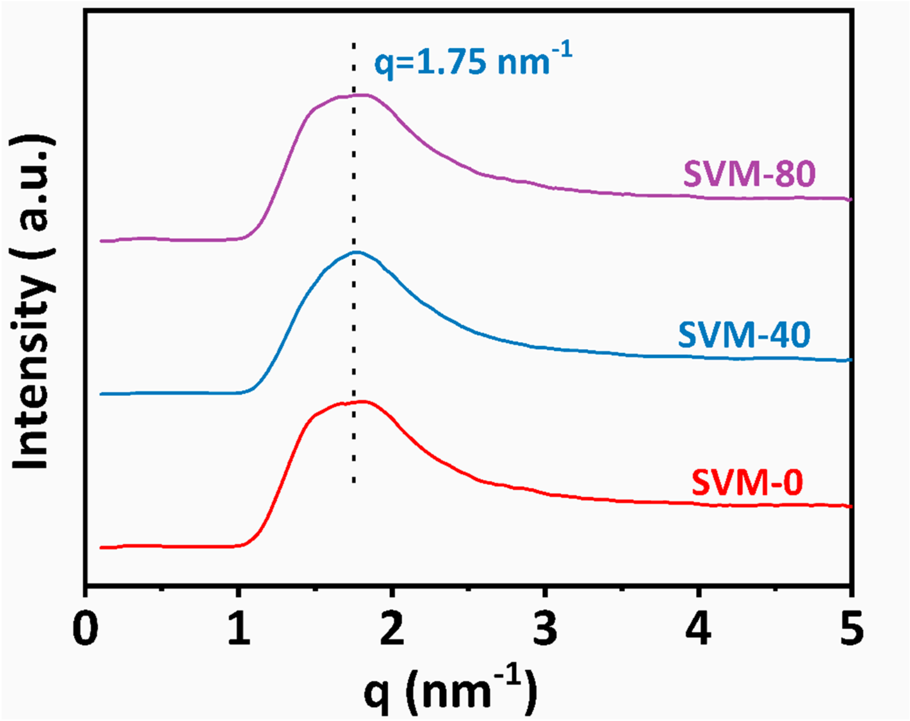

Figure S4. SAXS patterns of SVM-*x*.

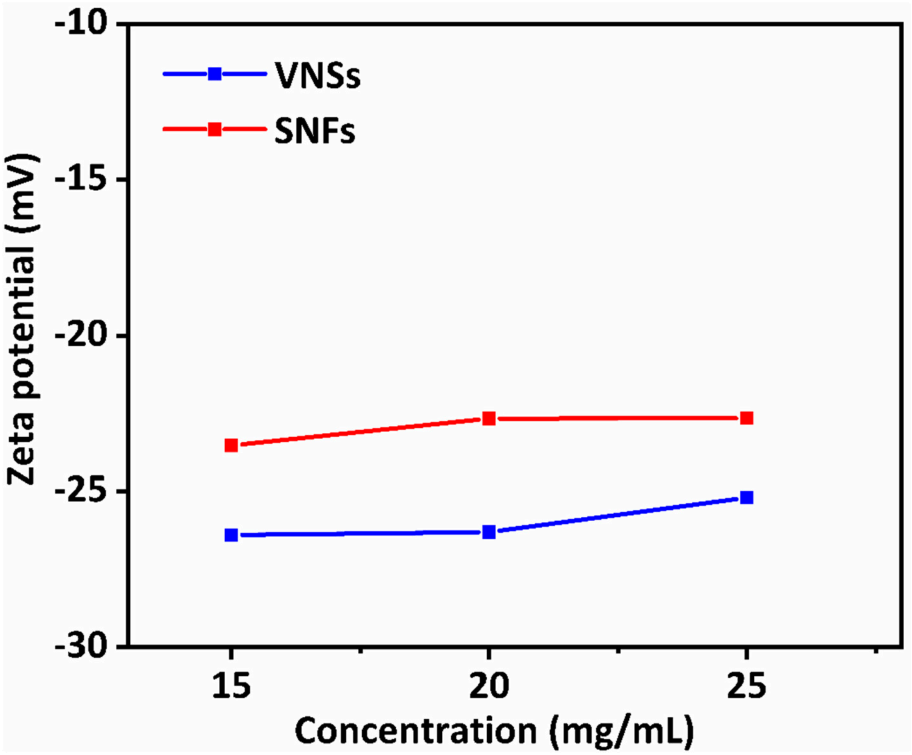

Figure S5. Concentration-dependent Zeta potentials of VNSs and SNFs dispersion.

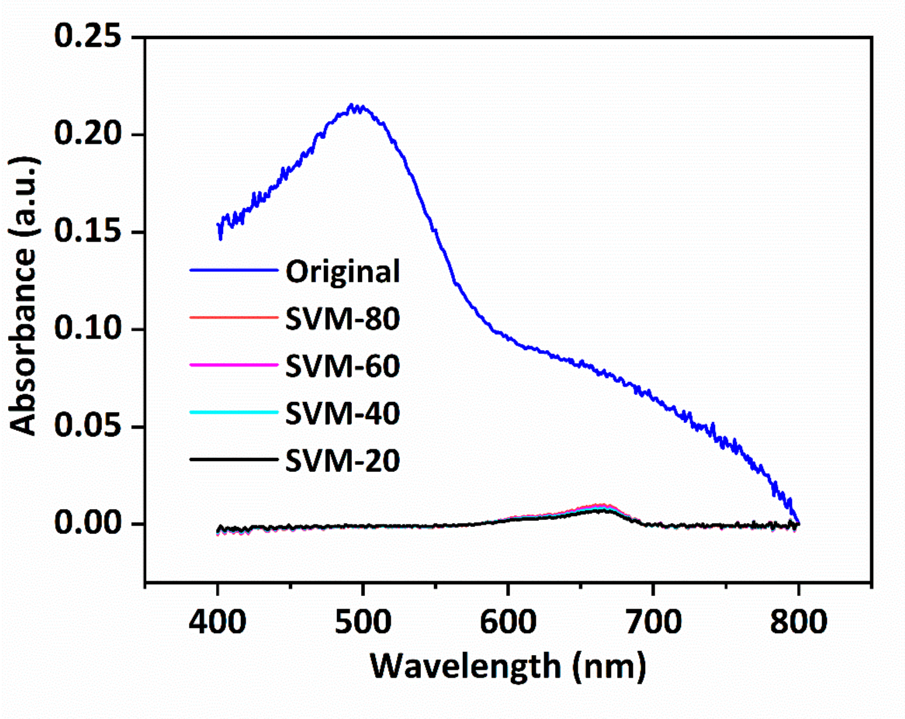

Figure S6. The UV-vis spectra of CR/NaCl solutions before and after treatment using SVM-x.

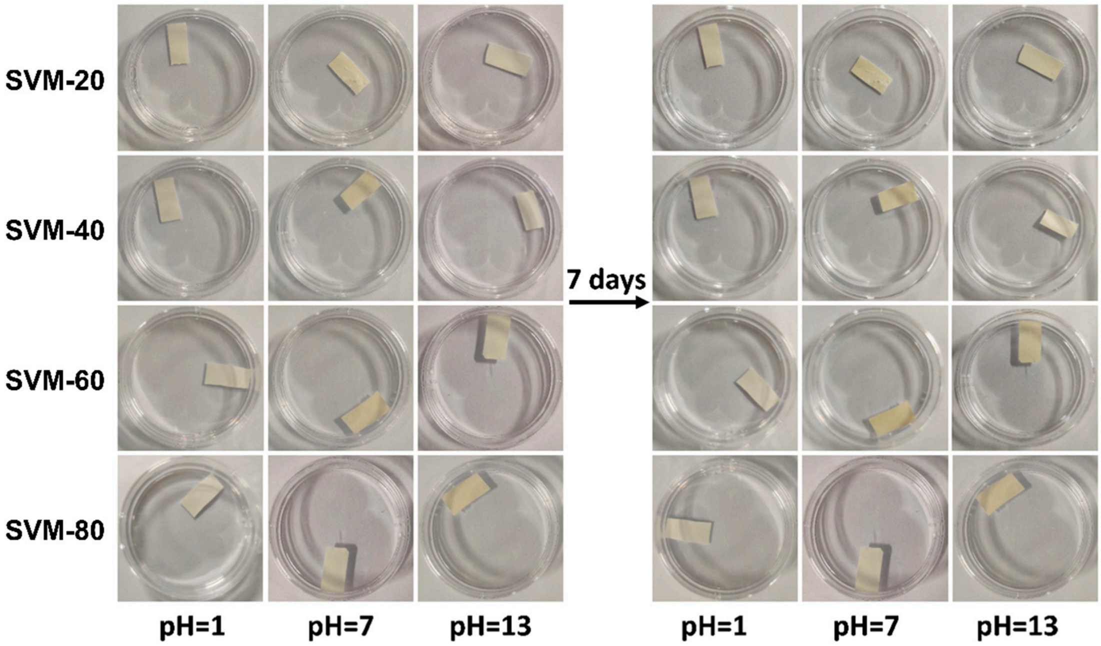

Figure S7. The digital photos of SVM-X before and after soaked in solutions of different pH.

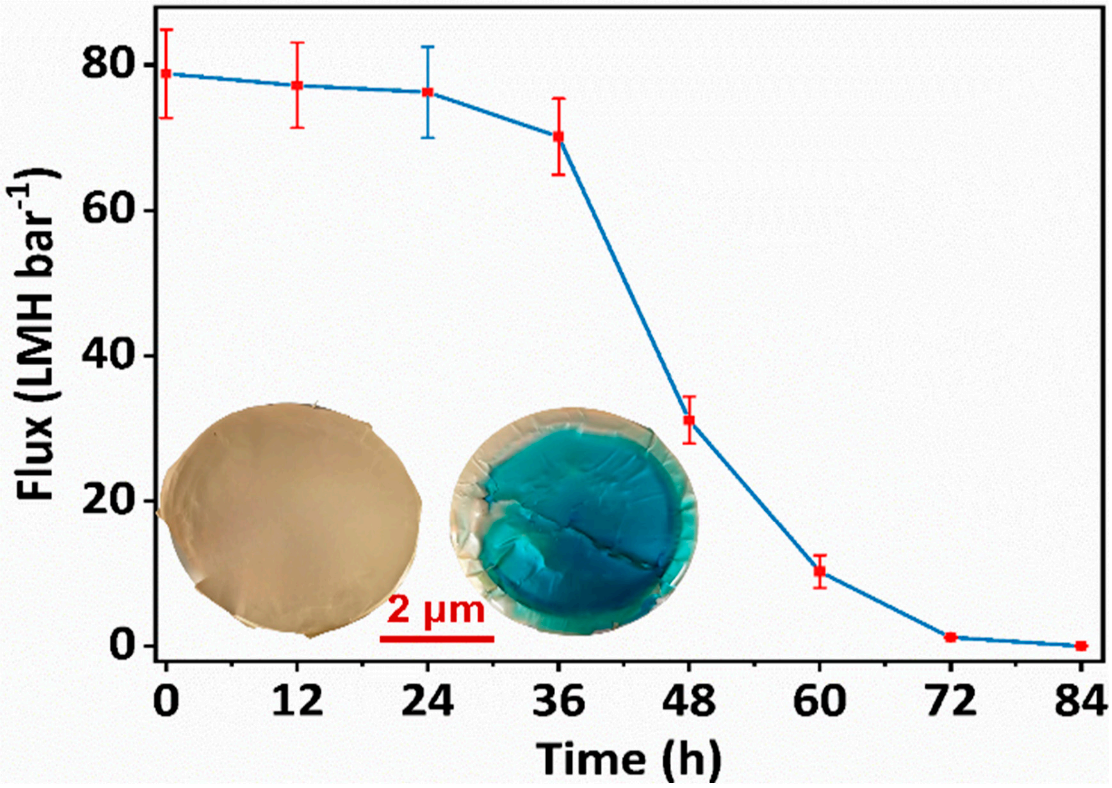

Figure S8. 84 h stability and dye/salt separation tests of SVM-80. The simulated wastewater contains 1 g L^{-1} NaCl and 10 mg L^{-1} MB for the tests. The insets show the digital images of SVM-80 before and after 84 h separation test.

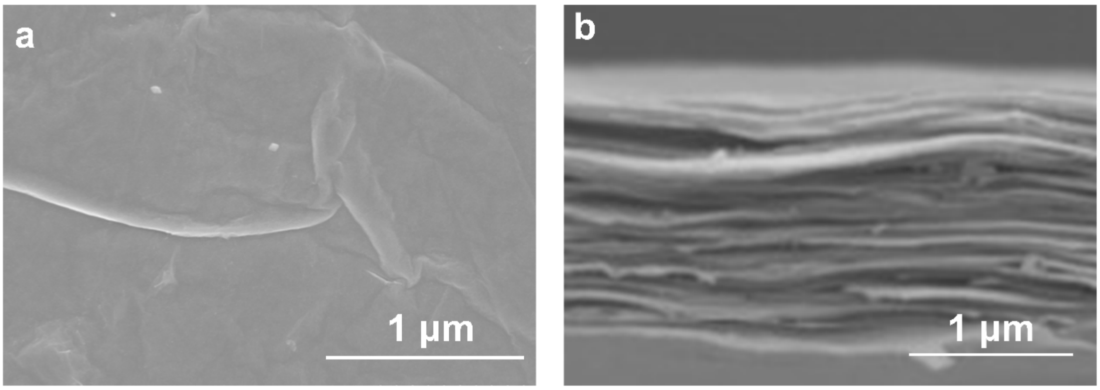

Figure S9. (a) Top-view SEM images of SVM-0 (pure vermiculite membrane) (b) Cross-section SEM images of SVM-0.

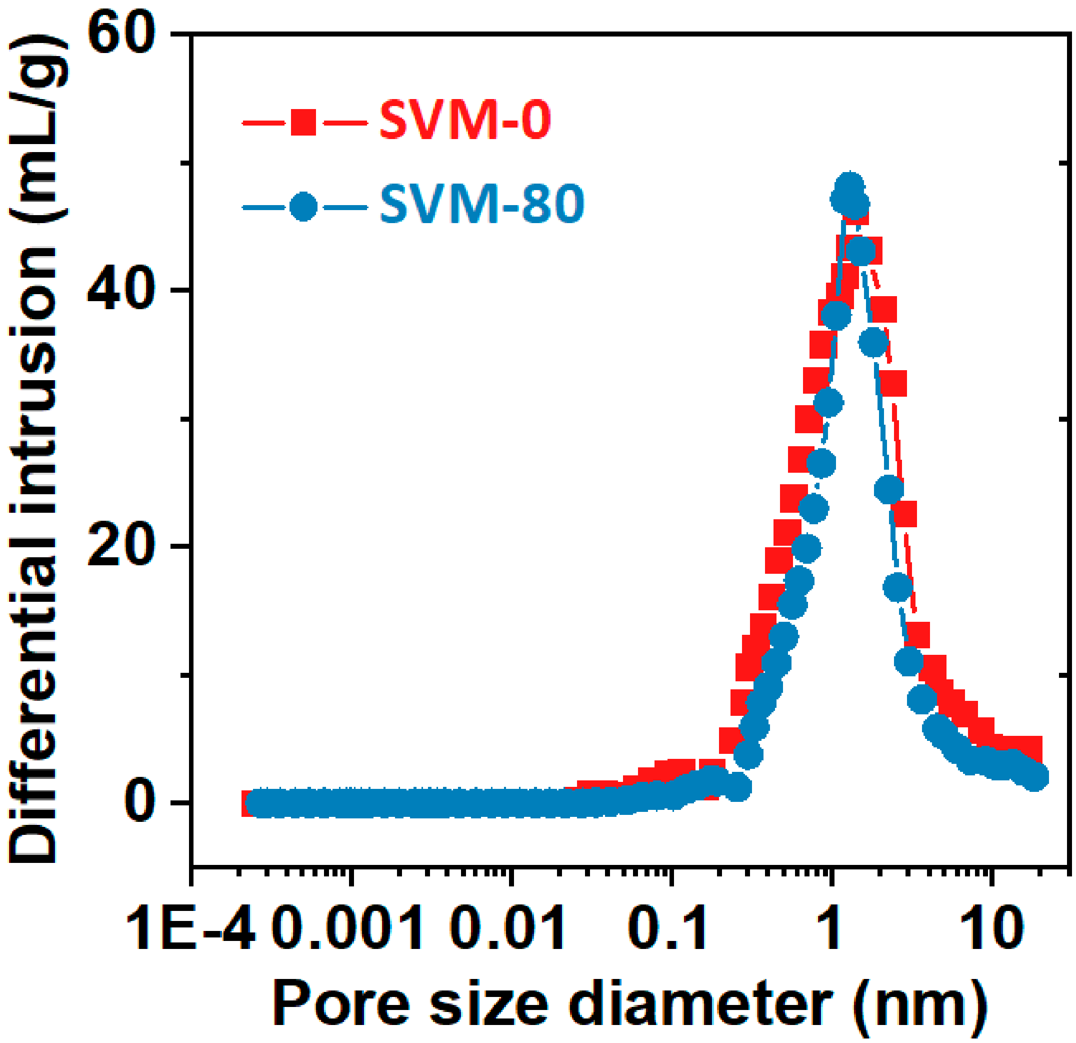

Figure S10. Pore size of SVM-0 and SVM-80 measured by the mercuric depressor.

Table S1 Comparison of salt retention capacity of other nanofiltration membranes. All the salts are 1 g/L NaCl.

Materials	NaCl rejection (%)	Flux (LMH bar−1)	Ref
MXene/PRGO	5.32	48.66	[1]
MXene/ZIF-8	7.88	40,85	[2]
MXene/CNT	23.52	10.81	[3]
GO/MoS2	43.27	10.27	[4]
PEI-modified GO/PAA/ PVA/GA	37.85	0.81	[5]
GO/HNTs	14.33	11.34	[6]
GO/NH2-Fe3O4	15.20	78.02	[7]
GOQDs TFN/TA	17.21	11.73	[8]
Vermiculite/Sepiolite	9.44	78.12	This work

Reference in the supporting material.

[1] S. Li, J. Lu, D. Zou, L. Cui, B. Chen, F. Wang, J. Qiu, T. Yu, Y. Sun, W. Jing, Constructing reduced porous graphene oxide for tailoring mass-transfer channels in ultrathin MXene (Ti3C2T) membranes for efficient dye/salt separation, Chemical Engineering Journal, 457 (2023) 141217.

[2] J. Li, L. Li, X. Li, L. Dong, Z. Wang, J. Shen, B. Van der Bruggen, Membranes with ZIF-8 regulated MXene nanosheet stacks for efficient molecular sieving, Desalination, 546 (2023) 116184.

[3] Y. Sun, D. Xu, S. Li, L. Cui, Y. Zhuang, W. Xing, W. Jing, Assembly of multidimensional MXene-carbon nanotube ultrathin membranes with an enhanced anti-swelling property for water purification, Journal of Membrane Science, 623 (2021) 119075.

[4] P. Zhang, J.-L. Gong, G.-M. Zeng, B. Song, W. Cao, H.-Y. Liu, S.-Y. Huan, P. Peng, Novel “loose” GO/MoS2 composites membranes with enhanced permeability for effective salts and dyes rejection at low pressure, Journal of Membrane Science, 574 (2019) 112-123.

[5] N. Wang, S. Ji, G. Zhang, J. Li, L. Wang, Self-assembly of graphene oxide and polyelectrolyte complex nanohybrid membranes for nanofiltration and pervaporation, Chemical Engineering Journal, 213 (2012) 318-329.

[6] L. Zhu, A porous graphene composite membrane intercalated by halloysite nanotubes for efficient dye desalination, Desalination, 420 (2017) 145-147.

[7] L. Dong, M. Li, S. Zhang, X. Si, Y. Bai, C. Zhang, NH2-Fe3O4-regulated graphene oxide membranes with well-defined laminar nanochannels for desalination of dye solutions, Desalination, 476 (2020) 114227.

[8] C. Zhang, K. Wei, W. Zhang, Y. Bai, Y. Sun, J. Gu, Graphene Oxide Quantum Dots Incorporated into a Thin Film Nanocomposite Membrane with High Flux and Antifouling Properties for Low-Pressure Nanofiltration, ACS Applied Materials & Interfaces, 9 (2017) 11082-11094.
